# Optical coherence tomography angiography biomarkers in a bi-monthly maintenance dosing aflibercept in patients with neovascular age-related macular degeneration

**DOI:** 10.1186/s12886-023-03039-4

**Published:** 2023-07-12

**Authors:** Jong Beom Park, Kiyoung Kim, Min Seok Kang, Eung Suk Kim, Seung-Young Yu

**Affiliations:** Department of Ophthalmology, Kyung Hee University Hospital, Kyung Hee University, 23, Kyungheedae-ro,, Dongdaemun-gu, Seoul 02447 Republic of Korea

**Keywords:** Age-related macular degeneration (AMD/ARMD), Optical coherence tomography angiography (OCTA), Swept-source optical coherence tomography angiography (SS-OCTA), Aflibercept, Macular neovascularization, Vessel density, Vessel length density

## Abstract

**Purpose:**

To evaluate the correlations between swept-source optical coherence tomography angiography (SS-OCTA) parameters and clinical outcomes in eyes with neovascular age-related macular degeneration (nAMD) administered a bimonthly intravitreal aflibercept regimen.

**Methods:**

This prospective, single-arm, interventional study enrolled 33 patients with treatment-naïve nAMD. The eyes received three monthly aflibercept injections followed by five bi-monthly regimens (total 50 weeks). The structural parameters including central subfield thickness (CST) and 5 mm pigment epithelial detachment (PED) volume and microvascular parameters including macular neovascularization (MNV) area, vessel density (VD), and vessel length density (VLD) were recorded every before and 1 week after treatment.

**Results:**

Patients who gained > 5 letters of best-corrected visual acuity (BCVA) from the baseline showed greater decreases in VD and VLD during the loading phase. Patients without recurrent or persistent fluid during the maintenance phase showed greater decreases in CST and 5 mm PED volume after the first injection. The decrease in mean VD during the loading phase was significantly correlated with the final BCVA (r = -0.820, *p* = 0.004). Moreover, the decrease in mean VLD during the loading phase was significantly correlated with the improvement in the final BCVA (r = -0.726, *p* = 0.017).

**Conclusions:**

The decrease in mean VD during the loading phase was significantly negatively correlated with the final BCVA at the last visit. The decrease in mean VLD during the loading phase, mean CST during the loading phase, and the improvement in final BCVA showed significant correlations. Therefore, early changes in OCTA microvascular and OCT structural parameters could help predict clinical outcomes in nAMD.

**Trial Registration:**

The trial was registered with the Clinical Research Information Service (CRIS), which joined the WHO International Clinical Trials Registry Platform (ICTRP) (Registration number: KCT0007375, Date of first trial registration: 10/06/2022).

## Introduction

Neovascular age-related macular degeneration (nAMD) is a leading cause of blindness in the elderly population [1]. nAMD is characterized by macular neovascularization (MNV), which is the proliferation of abnormal vessels in the choroid that can leak fluid [2, 3]. nAMD causes visual impairment due to damage to the retina, retinal pigment epithelium (RPE), and choriocapillaris [4–6].

The injection of anti-vascular endothelial growth factor (VEGF) is a proven treatment for nAMD, with successful results reported worldwide. Therefore, improvement and maintenance of visual acuity can be expected with anti-VEGF therapy. In terms of treatment for nAMD, three clinical protocols are widely accepted for intravitreal anti-VEGF injections in patients with MNV: *pro re nata*, treat-and-extend, and fixed regimens to reduce the treatment burden [7–10]. Typically, an initial intravitreal anti-VEGF injection is administered at a fixed monthly interval, and various trials have assessed the safety and efficacy of monthly or bi-monthly fixed regimens [4, 11–15]. Therefore, predicting the response to treatment and prognosis can help in deciding treatment protocols.

Until recently, fluorescein angiography (FA) and indocyanine green angiography (ICGA) were essential for the diagnosis, treatment, and monitoring of changes in neovascularization. This paradigm has changed since the clinical introduction of optical coherence tomography angiography (OCTA). The advantages of OCTA, the next generation of OCT technology, compared to FA and ICGA are the high-resolution, non-invasive in vivo visualization of the retinal microvasculature. Consequently, spectral-domain (SD) and swept-source (SS) OCTA have been used to image the choroidal vasculature [16]. SS-OCTA has advantages over SD-OCTA as it uses a longer wavelength and higher laser power, which increase RPE penetration, resulting in a high-resolution image of the underlying choriocapillaris layer [17].

Several OCTA studies have reported results related to the structural features of abnormal blood vessels in MNV lesions in patients with nAMD, both before and after treatment, including the characterization of microvascular morphologies such as seafan, tangled, medusa, and dead-tree [18, 19]. Additionally, OCTA studies have reported changes in OCTA biomarkers such as fractal dimension, lacunarity index, vessel density, flow deficits, vascular loop, and dark halo before and after treatment, serving as OCTA biomarkers [20–24]. Consequently, these OCTA studies have demonstrated the potential for qualitative and quantitative analysis of MNV lesions using OCTA. However, despite these efforts, OCTA studies have not yet been able to establish treatment guidelines based on OCTA findings, and OCTA microvascular parameter comparisons between different stages remain limited [18]. Therefore, clinical practice continues to rely on OCT structural parameters, such as intraretinal and subretinal fluid [18, 25, 26]. We aimed to determine the typical changes in MNV during the first year of intravitreal anti-VEGF treatment and whether there is a specific relationship between OCTA microvascular parameters and clinical outcomes in treatment-naïve nAMD patients with a fixed dosing regimen. Therefore, this study aimed to predict the long-term clinical outcomes of patients with nAMD and prospectively evaluate the anatomical changes in the MNV lesion using OCTA microvascular parameters and OCT structural parameters during the first year of bi-monthly intravitreal injections of fixed doses of aflibercept. We also analyzed the correlations between OCTA microvascular parameters of MNV activity, changes in OCT structural parameters and clinical outcomes in treatment-naïve patients with nAMD.

## Methods

The design of this prospective, single-arm interventional study was approved by the Institutional Review Board of Kyung Hee University Hospital (KHUH 2017-12-092) and adhered to the tenets of the Declaration of Helsinki. Informed consent was obtained from all patients before their study enrollment. The trial was registered with the Clinical Research Information Service (CRIS), which joined the WHO International Clinical Trials Registry Platform (ICTRP) (Registration number, KCT0007375, Date of first trial registration: 10/06/2022). Forty eyes of 40 patients were enrolled. The inclusion criteria were: (1) patients with active MNV confirmed by FA and ICGA or OCTA; (2) men and women ≥ 50 years of age who have signed the informed consent form; (3) treatment-naïve study eye; (4) no known active ocular or systemic diseases; and (5) patients who understand the process of this clinical trial, are cooperative, and are judged to be able to participate until the end of the clinical trial. The exclusion criteria applied to this study eye only and were as follows: (1) clinically significant disciform scar or macular atrophy; (2) active intraocular infection and inflammation; (3) known hypersensitivity to aflibercept or any excipients; (4) history of any ocular surgery within the past 3 months, including vitrectomy, glaucoma surgery, and corneal transplant surgery other than uncomplicated cataract surgery and refractive surgery within 3 months; (5) history of any retinal vascular diseases affecting visual acuity, including clinically significant diabetic retinopathy, diabetic macular edema and retinal vein occlusion; (6) history of intravitreal triamcinolone injection therapy within the past 3 months or intravitreal dexamethasone injection therapy within the past 6 months; (7) history of continuous use of inhaled or nebulized or topical steroid medications for more than 30 days within the past 3 months; (8) patients who have received intravitreal anti-vascular endothelial growth factor or panretinal photocoagulation treatment in the past; (9) diagnosis of cerebrovascular disease or myocardial infarction within the past year; (10) pregnant or breastfeeding women; and (11) patients currently participating in another clinical trial.

Three loading intravitreal aflibercept injections were administered each month. After the loading phase, five additional intravitreal aflibercept injections were administered every 8 weeks during the maintenance phase. The subjects were examined before every treatment and 1 week after every intravitreal injection to evaluate the early changes in the microvascular components of the MNV. During each of the 16 visits, the following ophthalmologic examinations were performed: best-corrected visual acuity (BCVA) using the Early Treatment Diabetic Retinopathy Study (ETDRS) visual chart, slit lamp examination, color fundus photography, spectral-domain OCT, and SS-OCTA. In this study, clinical outcomes were classified into functional outcomes and anatomical outcomes. The final BCVA at the last visit (week 49) and the improvement in the final BCVA during the entire study period (week 49 - week 0) were used as the functional outcomes. Furthermore, central subfield thickness (CST), 5 mm pigment epithelial detachment (PED) volume, presence of fluid, MNV area, vessel density (VD), and vessel length density (VLD) were defined as parameters for anatomical outcomes.

### Analysis of structural OCT and quantitative OCTA

In every evaluation, we performed a macular cube 512 × 128 scan of the Cirrus HD-OCT (Carl Zeiss Meditec, Dublin, CA) and a 6 × 6 mm scanning area centered on the fovea of the SS-OCTA (PLEX Elite 9000; Carl Zeiss Meditec, Dublin, CA). The PED volume in the central circle (5 mm in diameter) was calculated and processed using the built-in tools of the advanced RPE analysis algorithm of Cirrus HD-OCT. Based on the SS-OCTA images, the MNV area, VD, and VLD were analyzed as the OCTA microvascular parameters of MNV activity using ImageJ version 1.50 (National Institutes of Health, Bethesda, MD, USA). We analyzed the en face 6 × 6 mm SS-OCTA images using ImageJ. We extracted SS-OCTA images by selecting the top layer as the RPE and the bottom layer as the RPE fit mode. We manually edited the segmentation to remove projection artifacts for the MNV image analysis. The images were analyzed collaboratively by two trained graders using ImageJ, who reached a consensus during the evaluation process. The MNV area was manually outlined using a freehand selection tool and copied to a new black 600 × 600-pixel image. We reproduced a new MNV image using an adjustment tool to reduce artifacts and make the MNV image clearer for image analysis. The MNV area was calculated using the analysis measure tool in ImageJ. As previously described, VD was calculated after adjusting the brightness, contrast, and binarization of each image [17]. To evaluate VD, we considered all white pixels as vessels and black pixels as the background. The VD was calculated as the ratio of white pixels to the total pixels in the MNV area. VLD was evaluated using the skeletonize and vessel analysis tools in ImageJ. The associations of these parameters with the clinical response were assessed before treatment, 1 week after the first and third intravitreal injections of the loading phase, and the last intravitreal injection.

### Statistical analysis

Statistical analysis was performed using IBM SPSS Statistics for Windows, version 23.0 (IBM Corp., Armonk, NY, USA). Statistical significance was set at *p* < 0.05. Normality of the data distributions was assessed using the Shapiro–Wilk test. The χ2 test and Fisher’s exact test were used for comparing categorical data, which were presented as n. Mann–Whitney U tests were applied. Spearman correlation tests were used to estimate correlations between BCVA and CST, 5 mm PED volume, MNV area, VD, and VLD of the mean changes from baseline to 1 week after the third intravitreal injection during the loading phase. Fractions were used rather than simply subtracting the amounts of change in VD and VLD.

## Results

**Fig. 1:**
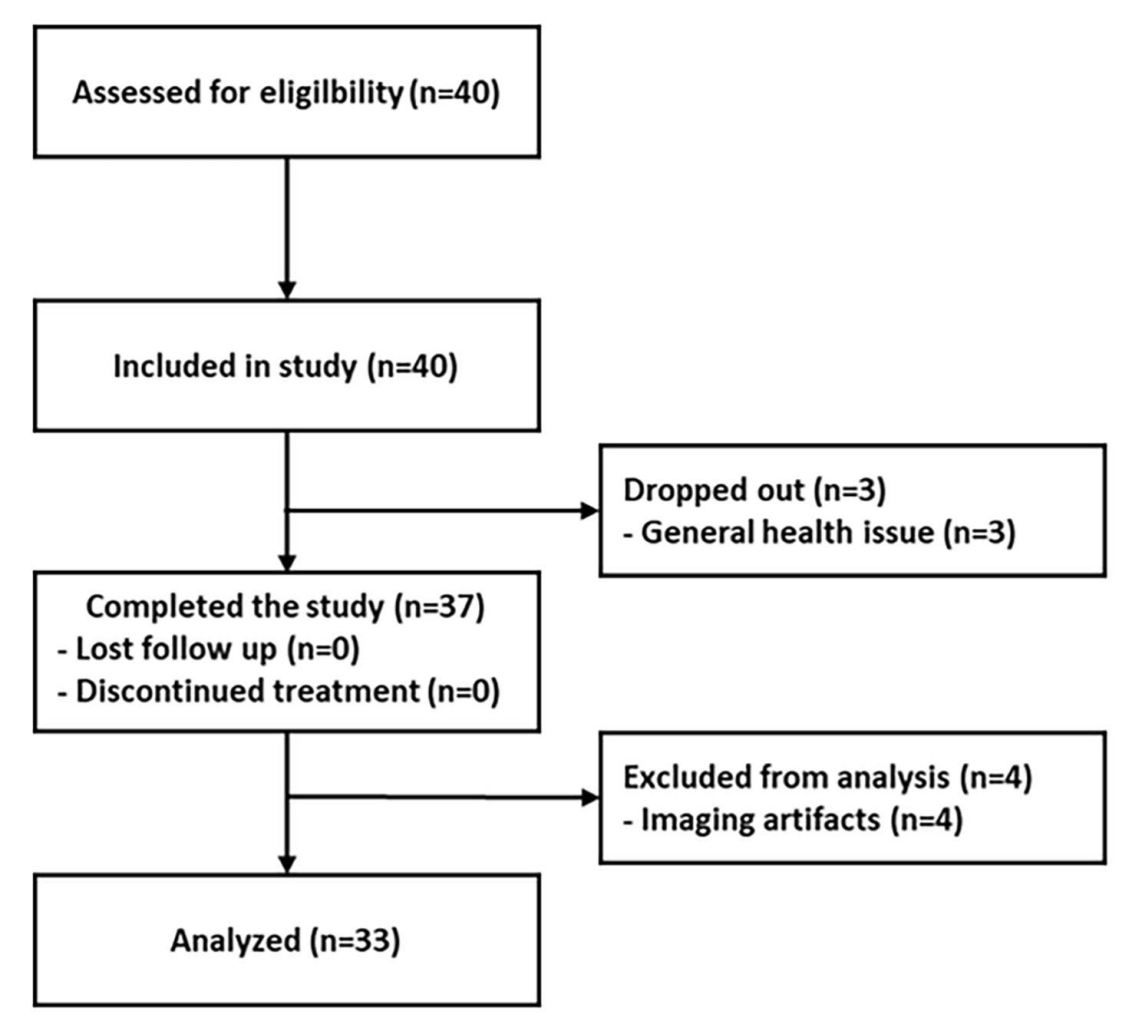
The flow diagram of the clinical trial showing the number of participants who were included, completed and analyzed in the study

While 40 eyes of 40 Korean patients were initially enrolled in the prospective study, three patients dropped out due to other unrelated general health issues. 37 patients successfully completed the 12-month study protocol. However, four patients had imaging artifacts in the ophthalmic imaging examination from at least one visit out of the 16 scheduled visits, making image analysis impossible. As a result, image analysis was performed on images obtained from 33 participants (Fig. 1). The demographic and clinical characteristics of the 33 participants are summarized in Table 1.

**Table 1:** Demographic and baseline OCT structural and OCTA microvascular parameter characteristics

Characteristics	
Number of eyes, n	33
Sex, n (%)	
Male	26 (78.8)
Female	7 (21.2)
Age (years)	72.38 ± 8.82
Diabetes mellitus, n (%)	12 (36.4)
Hypertension, n (%)	19 (57.6)
Pseudophakia, n (%)	4 (12.1)
Symptom duration (months)	3.65 ± 3.52
BCVA (ETDRS letters)	50.27 ± 23.57
CST (µm)	350.03 ± 110.09
5 mm PED volume (mm^3^)	0.65 ± 1.25
MNV area (mm^2^)	2.3722 ± 1.7167
Vessel density (%)	39.2 ± 7.7
Vessel length density (%)	11.96 ± 3.37

OCT = optical coherence tomography, OCTA = optical coherence tomography angiography, BCVA = best-corrected visual acuity, ETDRS = early treatment diabetic retinopathy study, CST = central subfield thickness, PED = pigment epithelial detachment, MNV = macular neovascularization

We analyzed the changes in visual acuity in two groups: one group in which 20 patients showed BCVA improvement of > 5 letters at the last visit compared to the first visit, and one group that did not show BCVA improvement. The two groups showed a statistically significant difference in the improvement of mean BCVA after the first injection (week 1 – week 0) and after the loading phase (week 9 –week 0). Among the OCTA microvascular parameters, the mean changes in VD and VLD between both groups differed significantly after the loading phase (week 9/week 0). Better BCVA at the last visit may be expected in patients showing an earlier greater decrease in VD and VLD during the loading phase (Table 2).

**Table 2:** Comparisons of early OCT structural and OCTA microvascular parameters between patients with and without BCVA improvement

	Patients with BCVA improvement (n = 20)*	Patients without BCVA improvement (n = 13)	*p*
Age (years)	71.70	71.77	0.676
Diabetes mellitus (n)	8	4	0.719
Hypertension (n)	11	8	0.710
Pseudophakia (n)	2	2	1.000
Symptom duration (months)	3.06	4.56	0.392
Mean change in BCVA (week 1–week 0) (ETDRS letters)	11.90	2.08	0.003
Mean change in BCVA (week 9–week 0) (ETDRS letters)	16.60	2.38	0.002
Mean change in CST (week 1–week 0) (µm)	-64.80	-41.23	0.147
Mean change in CST (week 9–week 0) (µm)	-126.00	-104.46	0.207
Mean change in 5 mm PED volume (week 1–week 0) (mm^3^)	-0.22	-0.17	0.194
Mean change in 5 mm PED volume (week 9–week 0) (mm^3^)	-0.42	-0.44	0.235
Mean change in MNV area (week 1–week 0) (mm^2^)	-0.94	-0.82	0.610
Mean change in MNV area (week 9–week 0) (mm^2^)	-1.06	-1.10	0.914
Mean change in vessel density (week 1/week 0) (%)	0.84	0.96	0.762
Mean change in vessel density (week 9/week 0) (%)	0.78	1.13	0.038
Mean change in vessel length density (week 1/week 0) (%)	0.82	0.89	0.610
Mean change in vessel length density (week 9/week 0) (%)	0.81	1.08	0.019

OCT = optical coherence tomography, OCTA = optical coherence tomography angiography, BCVA = best-corrected visual acuity, ETDRS = early treatment diabetic retinopathy study, CST = central subfield thickness, PED = pigment epithelial detachment, MNV = macular neovascularization

* BCVA improvement of more than five ETDRS letters

We also compared two groups of patients with and without recurrent or persistent fluid during the maintenance phase from week 10 to week 49. 20 patients showed no fluid during the maintenance phase, while 13 patients showed recurrent or persistent fluid, subretinal fluid (SRF), or intraretinal fluid (IRF) during the same period. Both groups showed statistically significant differences in the decrease in mean CST and 5 mm PED volume after the first aflibercept injection. Patients with an early larger decrease in CST and 5 mm PED volume showed no fluid during the maintenance phase (Table 3).

**Table 3:** Comparisons of early OCT structural and OCTA microvascular parameters between patients with and without recurrent or persistent fluid during the maintenance phase

	Without fluid (n = 20)	Recurrent or Persistent fluid (n = 13)	*p*
Age (years)	72.70	70.23	0.413
Diabetes mellitus (n)	6	6	0.465
Hypertension (n)	12	7	0.727
Pseudophakia (n)	1	3	0.276
Symptom duration (months)	3.55	3.81	0.957
Mean change in BCVA (week 1–week 0) (ETDRS letters)	9.65	5.54	0.334
Mean change in BCVA (week 9–week 0) (ETDRS letters)	11.40	10.38	0.624
Mean change in CST (week 1–week 0) (µm)	-72.80	-28.92	0.027
Mean change in CST (week 9–week 0) (µm)	-139.65	-83.46	0.194
Mean change in 5 mm PED volume (week 1–week 0) (mm^3^)	-0.32	-0.02	0.048
Mean change in 5 mm PED volume (week 9–week 0) (mm^3^)	-0.58	-0.19	0.730
Mean change in MNV area (week 1–week 0) (mm^2^)	-0.59	-1.10	0.257
Mean change in MNV area (week 9–week 0) (mm^2^)	-0.82	-1.25	0.914
Mean change in vessel density (week 1/week 0) (%)	0.73	0.99	0.171
Mean change in vessel density (week 9/week 0) (%)	0.91	0.93	0.610
Mean change in vessel length density (week 1/week 0) (%)	0.76	0.91	0.352
Mean change in vessel length density (week 9/week 0) (%)	0.92	0.91	0.914

OCT = optical coherence tomography, OCTA = optical coherence tomography angiography, BCVA = best-corrected visual acuity, ETDRS = early treatment diabetic retinopathy study, CST = central subfield thickness, PED = pigment epithelial detachment, MNV = macular neovascularization

The correlations between early OCT structural parameters, OCTA microvascular parameters, and functional outcomes were statistically analyzed. Regarding functional outcomes, the decrease in mean VD during the loading phase was significantly negatively correlated with the final BCVA at the last visit. (r = -0.820, *p* = 0.004) In addition, the decrease in mean VLD during the loading phase (r = -0.726, *p* = 0.017), mean CST during the loading phase (r = -0.862, *p* = 0.001), and the improvement in final BCVA (week 49 – week 0) were significantly correlated (Table 4).

**Table 4:** Correlations between BCVA as a functional outcome and early OCT structural and OCTA microvascular parameters

	Mean change in CST (week 9–week 0)	Mean change in 5 mm PED volume (week 9–week 0)	Mean change in MNV area (week 9–week 0)	Mean change in vessel density (week 9/week 0)	Mean change in vessel length density (week 9/week 0)
Final BCVA (week 49)	r = -0.024 *p* = 0.947	r = 0.065 *p* = 0.859	r = 0.502 *p* = 0.140	r = -0.820 *p* = 0.004	r = -0.275 *p* = 0.441
Improvement in final BCVA (week 49–week 0)	r = -0.862 *p* = 0.001	r = -0.529 *p* = 0.116	r = -0.191 *p* = 0.598	r = -0.542 *p* = 0.106	r = -0.726 *p* = 0.017

OCT = optical coherence tomography, OCTA = optical coherence tomography angiography, BCVA = best-corrected visual acuity, CST = central subfield thickness, PED = pigment epithelial detachment, MNV = macular neovascularization

r = Spearman correlation coefficient

**Fig. 2:**
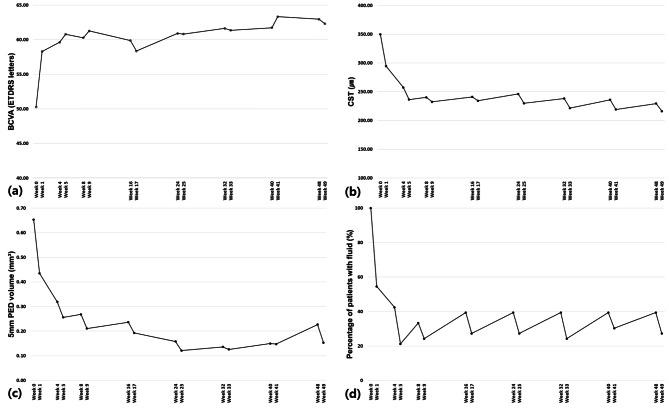
Changes in functional and anatomical outcomes of 33 eyes over 50 weeks. Three intravitreal aflibercept injections were administered every 4 weeks (weeks 0, 4, and 8) during the initial 3 months of the loading phase. After the loading phase, five intravitreal aflibercept injections were administered every 8 weeks (weeks 16, 24, 32, 40, and 48) during the maintenance phase. **(a)** Mean best-corrected visual acuity (BCVA) as the functional outcome. **(b)** Mean central subfield thickness (CST) as an optical coherence tomography (OCT) structural parameter. **(c)** Mean 5 mm pigment epithelial detachment (PED) volume as an OCT structural parameter **(d)** Percentage of patients with fluid as an OCT structural parameter

**Fig. 3:**
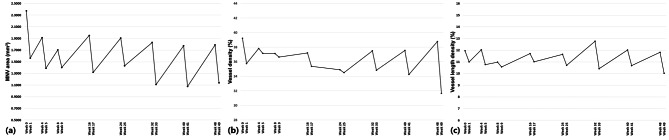
Changes in functional and anatomical outcomes of 33 eyes over 50 weeks. Three intravitreal aflibercept injections were administered every 4 weeks (weeks 0, 4, and 8) during the initial 3 months of the loading phase. After the loading phase, five intravitreal aflibercept injections were administered every 8 weeks (weeks 16, 24, 32, 40, and 48) during the maintenance phase. **(a)** Mean macular neovascularization (MNV) area as an optical coherence tomography angiography (OCTA) microvascular parameter, **(b)** Mean vessel density as an OCTA microvascular parameter. **(c)** Mean vessel length density as an OCTA microvascular parameter

The prospective changes in mean BCVA, CST, 5 mm PED volume, presence of fluid, MNV area, VD, and VLD at every visit are shown in Figs. 2 and 3. The mean BCVA improved during the loading phase, particularly after the first injection. The BCVA gain was maintained throughout the year (Fig. 2(a)). As shown in Fig. 2(b), the mean CST was reduced, particularly during the first month, with a pattern similar to that of BCVA. While slight increases were observed after the injections, the reduction in mean CST was generally maintained. Figure 2(c) shows the mean 5 mm PED volume, which decreased during the loading and early maintenance phases. Figure 2(d) presents the percentage of patients with fluid at each visit. After an initial decrease in fluid within the first month, the changes during the maintenance phase were overall similar to the changes in CST as shown in Fig. 2(b). Figure 3(a) shows the changes in the mean MNV area during the year of bi-monthly fixed dosing. This area decreased 1 week after injection and then increased again. Figure 3(b) shows the mean changes in VD. The pattern of changes in VD was similar to that for the mean 5 mm PED volume during the first 6 months and the mean MNV area after the other 6 months. In addition, the VD decreased continuously even during the maintenance period. Figure 3(c) shows the changes in VLD. The continuous decrease was more prominent in VLD.

## Discussion / conclusion

The advantage of OCTA is the non-invasive high-resolution visualization of MNV microvasculature and morphological information; therefore, OCTA can be repeated easily at follow-up visits in patients with nAMD. OCTA can be used to detect MNV or other retinal lesions without dye injection for FA and ICGA. OCTA has been evaluated as an alternative to FA and ICGA [27]. Previous studies reported that OCTA can detect MNV blood flow with the same sensitivity as FA [28] and visualize the area of MNV more accurately than ICGA [29, 30]. Lumbroso et al. reported an earlier reduction in type 2 MNV area on OCTA and pruning of smaller vessels immediately after intravitreal anti-VEGF injection in five eyes at 24 hours [31]. In addition, Mastropasqua et al. observed no reduction in type 1 MNV size on OCTA after aflibercept injection in 15 naïve eyes [32]. While these findings analyzed the effect of anti-VEGF therapy after intravitreal injection with OCTA, they were not a long-term prospective study. Therefore, we prospectively analyzed the correlation between OCTA microvascular biomarkers of MNV activity and changes in MNV area, VD, VLD by SS-OCTA, and OCT structural biomarkers such as CST, 5 mm PED volume with clinical outcomes in patients with nAMD undergoing a fixed dosing regimen for 50 weeks.

Unlike previous studies, the patients in the present study were examined before every treatment and 1 week after every intravitreal injection. This study protocol made it possible to evaluate the early changes in the microvascular and structural components of MNV lesions. The fractions of the changes in VD and VLD were used for more accurate evaluation compared to subtracting the amount of change. Since MNV area does not change with changes in VD and VLD, or MNV area can change without changes in VD and VLD after anti-VEGF treatment, the accurate determination of changes in VD and VLD may not be reflected simply by subtraction. Therefore, to more accurately evaluate the changes in VD and VLD, naive VD and VLD values were used as the denominator, while the VD and VLD values measured after treatment were used as the numerator.

We observed a significant difference in the mean change in VD and VLD after the loading phase (week 9/week 0) between the BCVA improvement and no-BCVA improvement groups (Table 2). However, the MNV area was not an OCTA microvascular parameter related to BCVA improvement. Therefore, the greater the amounts of early changes in the VD and VLD after the loading phase, which is the OCTA microvascular parameter, or the amount of change in BCVA improvement after the first injection and after the loading phase, the better the visual prognosis. Thus, the results of this study demonstrated the prediction of the final visual acuity of patients with nAMD undergoing a fixed dosing regimen after treatment. Regarding anatomical outcomes, the presence of unresolved SRF and IRF as fluid or recurrent fluid after intravitreal injection in nAMD patients is important. Compared to the recurrent fluid group, the patient group maintained without fluid during the maintenance phase showed statistically greater decreased CST and 5 mm PED volume after the first intravitreal injection, with no difference in OCTA microvascular parameters (Table 3). Based on these results, it is not possible to suggest that only OCTA parameters are related to visual acuity. Similarly, it is not appropriate to discuss that OCT parameters are related to fluid presence. The reason is that, as shown in Table 4, both OCTA and OCT parameters, such as decreases in mean CST, VD, and VLD during the loading phase, demonstrated significant correlations with both the final BCVA and the improvement in final BCVA. Therefore, the prediction of the clinical outcomes of patients with nAMD should consider both the early changes in OCTA microvascular parameters and OCT structural parameters, including the loading phase.

**Fig. 4:**
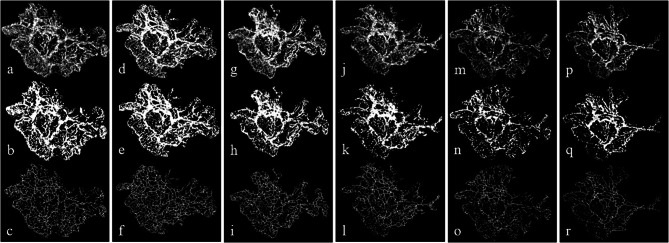
Analysis of a series of swept-source optical coherence tomography angiography (SS-OCTA) images from an 84-year-old man with neovascular age-related macular degeneration (nAMD). Eight intravitreal aflibercept injections were performed at weeks 0, 4, 8, 16, 24, 32, 40, and 48. The associations of optical coherence tomography angiography (OCTA) microvascular parameters with the clinical response were assessed before the intravitreal injections, 1 week after the first and third intravitreal injections during the loading phase, and the last intravitreal injection (weeks 0, 1, 8, 9, 48, and 49). The small branching blood vessels of macular neovascularization (MNV) lesions showed attenuation after the intravitreal injections. However, the persistence, hyperintense signal of the core feeder, and main large vessel trunk changes appeared to be insignificant in the series of SS-OCTA images. Top row: MNV area analysis of the MNV lesion. The en face 6 × 6 mm SS-OCTA images were analyzed to calculate the MNV area using ImageJ software. Middle row: Vessel density analysis of the MNV lesion. The vessel density was calculated after adjusting the brightness, contrast, and binarization of each image. Bottom row: Vessel length density analysis of the MNV lesion. The vessel length density was evaluated using the skeletonize and vessel analysis tools in ImageJ software. (a–c) Week 0. (d–f) Week 1. (g–i) Week 8. (j–l) Week 9. (m–o) Week 48. (p–r) Week 49

The analysis of OCTA images in this prospective study demonstrated a series of changes in MNV microvasculature before and after intravitreal aflibercept injection (Fig. 4). The changes in MNV microvasculature observed by OCTA included the change to attenuation of small branching blood vessels of MNV lesions after aflibercept injection; however, the persistence, perfusion, hyperintense signal of the core feeder, and main large vessel trunk changes were not significant. Our findings are similar to those of previous studies reporting that the exudative activity of MNV lesions includes the presence of small vessels, peripheral arcades, and a perilesional halo sign in the neovascular network before anti-VEGF treatment [19, 31, 33–35]. After treatment, subtle small vessel attenuation or pruning after anti-VEGF injections and vascular re-proliferation occur; therefore, MNV lesions regain a similar morphology to that before treatment [19, 36, 37]. The results of the present study suggest that VD and VLD, which reflect changes in small branch vessels rather than MNV area, were more associated with clinical outcomes in patients with nAMD after anti-VEGF treatment due to vascular attenuation and MNV lesion reproliferation (Table 2). This supports the fact that anti-VEGF injection modifies the vascular net and suggests that it agrees with Spaide’s abnormalization theory of MNV treated using a recurrent intravitreal anti-VEGF injection [38]. Moreover, the changes in the OCTA series images may correspond to decreases in VD and VLD as OCTA microvascular biomarkers. This was confirmed by the graphs of changes in MNV area, VD, and VLD (Fig. 3). That is, the smallest values of OCTA microvascular parameters measured 1 week after aflibercept injection all demonstrated a tendency to increase over time and were highest immediately before aflibercept injection. Therefore, VD and VLD rather than the MNV area play important roles in BCVA in patients with nAMD. Furthermore, the analysis of various OCTA biomarkers such as VD or VLD that can be identified by SS-OCTA in this study and OCT structural parameters showed the potential for proactive treatment by detecting these markers before the recurrence of SRF or IRF in patients with MNV, which appears to be a predictive factor for better visual prognosis. Thus, further studies on OCTA biomarkers in patients with MNV are needed.

One of the limitations of this study was its small sample size. Furthermore, we did not register the study specifically for patients with a particular type of MNV. Therefore, we were unable to analyze the structural and microvascular parameters according to MNV types. Another limitation was the current OCTA accuracy of the segmentation algorithms. To compensate for the limitation of OCTA segmentation accuracy, we performed manual segmentation at every visit for all patients; however, accurate manual segmentation of the MNV lesions was not possible. In addition, motion artifacts are due to the limitations of the current OCTA technology, which can result in low image quality and less accurate analysis of MNV lesions. The last limitation of this study was the use of ImageJ software. Because the quantitative analysis of MNV lesions and OCTA biomarkers using ImageJ was entirely manual, there may be problems with accuracy and reproducibility. For these reasons, this study may not have sufficient power to analyze OCTA microvascular parameters in patients with nAMD.

Recently, studies have presented findings on the potential of utilizing OCTA for qualitative and quantitative analysis of MNV lesions in nAMD patients. Research is underway on dense B-scan OCTA and high-resolution B-scan OCTA, and as OCTA technology improves, we can expect a reduction in noise, speckle, and projection artifacts [39]. Moreover, the research and development of deep learning algorithms are expected to enhance the accuracy of automated segmentation protocols and improve the overall image quality [40, 41]. Ongoing studies involve training AI with OCTA images to generate new retinal flow maps from structural OCT images [42]. These advancements instill anticipation for future OCTA technologies that will overcome the limitations associated with current OCTA methods.

In conclusion, the results of our prospective study demonstrated that OCTA is a promising new imaging modality that provides a noninvasive, dye-free, and reproducible method to analyze MNV lesions. OCTA analysis of the early microvasculature and morphological changes of MNV lesions and OCTA microvascular biomarkers can help predict the response to treatment with intravitreal anti-VEGF injections and establish an effective treatment plan.

## Data Availability

The datasets used and/or analyzed during the current study are available from the corresponding author on reasonable request.
